# Angioimmunoblastic T-cell lymphoma presenting as giant kidneys: a case report

**DOI:** 10.4076/1752-1947-3-9258

**Published:** 2009-09-14

**Authors:** Ori Argov, Gideon Charach, Moshe Weintraub, Alexander Shtabsky

**Affiliations:** 1 Department of Internal Medicine "C", Tel-Aviv Sourasky Medical Center, 6 Weizman St, Tel Aviv 64239, Israel, and The Sackler Faculty of Medicine, Tel-Aviv University, Tel-Aviv, Israel; 2 Pathology Institute, Tel-Aviv Sourasky Medical Center, 6 Weizman St, Tel Aviv 64239, Israel, and The Sackler Faculty of Medicine, Tel-Aviv University, Tel-Aviv, Israel

## Abstract

**Introduction:**

Angioimmunoblastic T-cell lymphoma is a rare form of tumor of the lymph nodes or lymphoid tissue. In this report we describe an unusual presentation of angioimmunoblastic T-cell lymphoma consisting of giant kidneys with no nephrotic syndrome.

**Case presentation:**

A 46-year-old Arabic man from Gaza was hospitalized in our ward due to abdominal pain and a weight loss of 20 kg during the preceding two months. The results of the physical examination and laboratory tests raised the possibility of neoplastic disease. A computerized tomographic scan of the abdomen showed huge kidneys, and a kidney biopsy showed infiltration by lymphocytes and eosinophils. The genetic examination revealed T-cell lymphoma. Diagnosis was made by a lymph node biopsy, which shows typical findings of angioimmunoblastic T-cell lymphoma.

**Conclusions:**

Angioimmunoblastic T-cell lymphoma can present with huge kidneys without nephrotic syndrome.

## Introduction

Angioimmunoblastic T-cell lymphoma is rare, occurring in only 1% of all cases of lymphoma. It is characterized by the loss of lymphoid architecture, with a pleomorphic cellular infiltrate and proliferation of small blood vessels. Patients usually present with B symptoms (weight loss, sweating and fever), generalized lymphadenopathy, skin rash, polyclonal hypergammaglobulinemia, autoimmune disorders (for example, Coombs-positive hemolytic anemia), or infections. Diagnosis can be made by a lymph node biopsy which will show infiltration of small lymphocytes, plasma cells, immunoblasts, histiocytes, and often eosinophils. The malignant cells are CD4+ αβT cells with TCR β and γ rearrangements that may express CD10 and Bcl-6. Treatment is through doxorubicin-based regimens. We report the case of a man who sought medical advice due to weight loss and abdominal pain, and whose unusual presentation of angioimmunoblastic T-cell lymphoma consisted of giant kidneys with no nephrotic syndrome.

## Case presentation

A 46-year-old Arabic man from Gaza was hospitalized in our ward due to abdominal pain and a weight loss of 20 kg during the preceding two months. His medical history did not contribute any useful information for the diagnosis and he denied fever or night sweats. His physical examination yielded normal results except for diffuse abdominal tenderness. His body temperature was 37.5°C during the first few days of hospitalization. Laboratory tests showed marked eosinophilia (30%), hyperglobulinemia (72 mg/l), and mild renal dysfunction (creatinine 1.7 mg/dl). There was no anemia (hemoglobin 13 mg/dl), leukocytosis, disturbance of liver function or elevation of C-reactive protein levels. Blood and urine cultures tested negative as did serologic tests for hepatitis, *Rickettsiae* and other zoonotic infections. PPD (tuberculin) tests were negative (twice) as was an HIV test, and we ruled out parasitic infection. The results of a complete panel of laboratory examinations for autoimmune diseases came back negative, and connective tissue disease was ruled out as well. A 24-hour urine collection ruled out nephrotic syndrome, and a microscopic examination of the urine was normal. The hyperglobulinemia was found to be polyclonal so the possibility of multiple myeloma was excluded.

**Figure 1:**
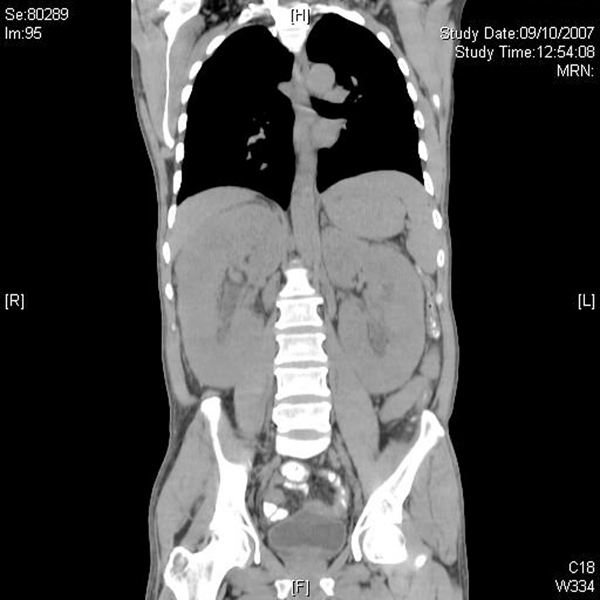
**Abdominal computerized tomographic scan showing kidneys measuring 22 cm in length**.

**Figure 2:**
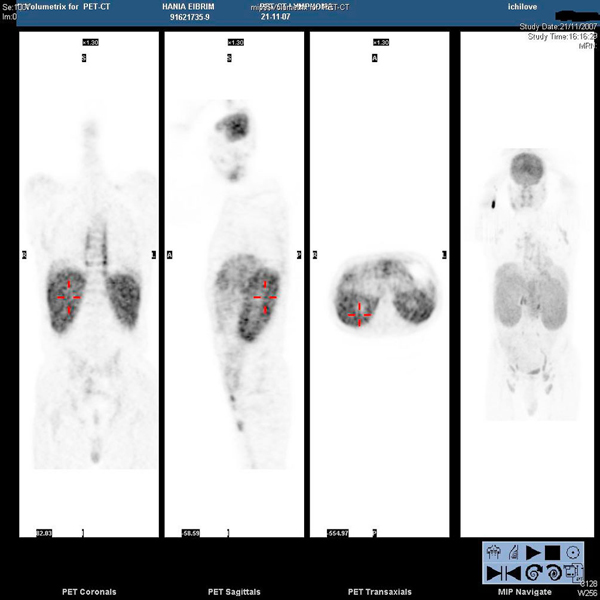
**A positron emission tomographic scan showed a diffuse nodular lymphoproliferative disease involving giant kidneys**.

**Figure 3:**
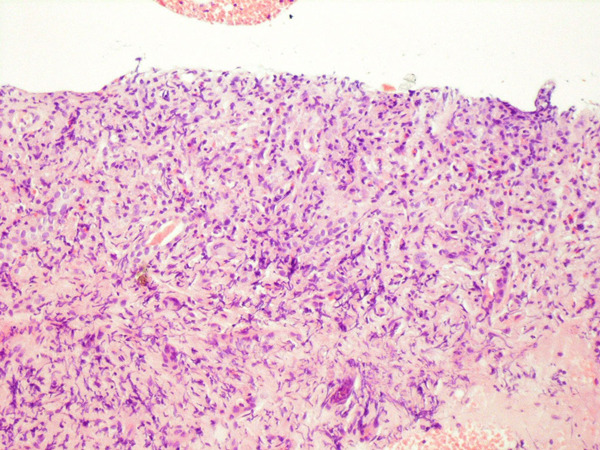
**Renal biopsy shows parenchyma effacement by the diffuse leukocytic infiltrate**.

**Figure 4:**
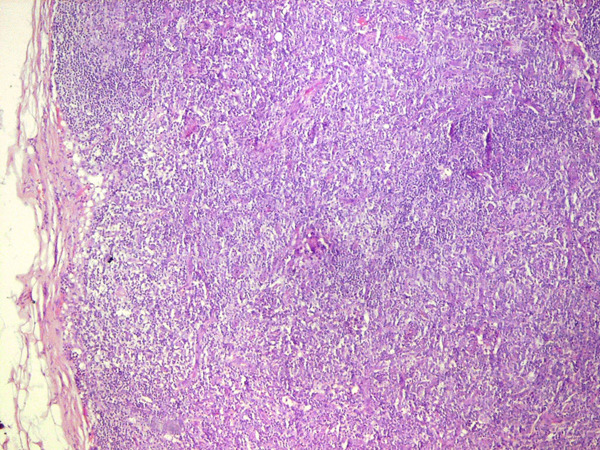
**Biopsy of a lymph node shows obliteration of the node architecture**.

**Figure 5:**
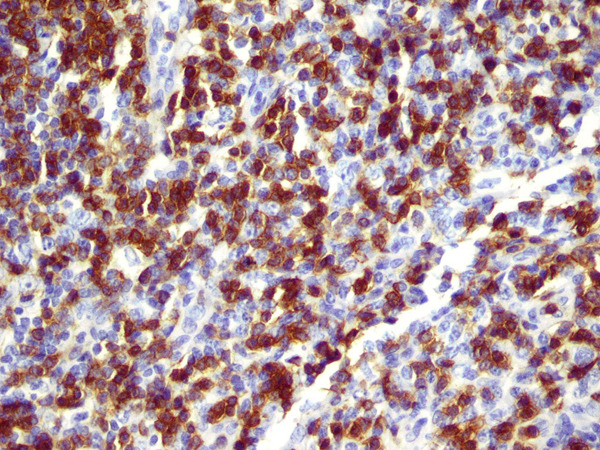
**Lymph node biopsy shows CD3 positive infiltrate**.

At this point, the combination of weight loss, eosinophilia and hyperglobulinemia raised the possibility of neoplastic disease. A computerized tomographic (CT) scan of the abdomen (Figure 1) showed huge kidneys measuring 22 cm in length. Numerous lymph nodes were found to be enlarged in the mediastinum, inguinal area and along the aorta. A positron emission tomographic CT (PET-CT) scan showed a diffuse nodular lymphoproliferative disease above and below the diaphragm, involving giant kidneys (Figure 2). A kidney biopsy showed effacement of the renal structure by diffuse leukocytic infiltrate, represented mostly by elongated cells with marked artifactual changes (Figure 3). A polymerase chain reaction analysis of the gamma T-cell receptor rearrangement showed monoclonality of the T cells, which raised the possibility of T-cell lymphoma. Infiltration by lymphocytes stained mostly for CD3 (T lymphocytes). A biopsy of an inguinal lymph node was remarkable for obliteration of the node architecture (Figure 4). The paracortical area was infiltrated by cells that were positive for CD3 and CD4 (Figure 5). Bone marrow biopsy showed eosinophilia without lymphatic aggregates. The constellation of eosinophilia, hyperglobulinemia, generalized lymphadenopathy, giant kidneys and the findings in the lymph node biopsy were consistent with the diagnosis of angioimmunoblastic T-cell lymphoma.

**Figure 6:**
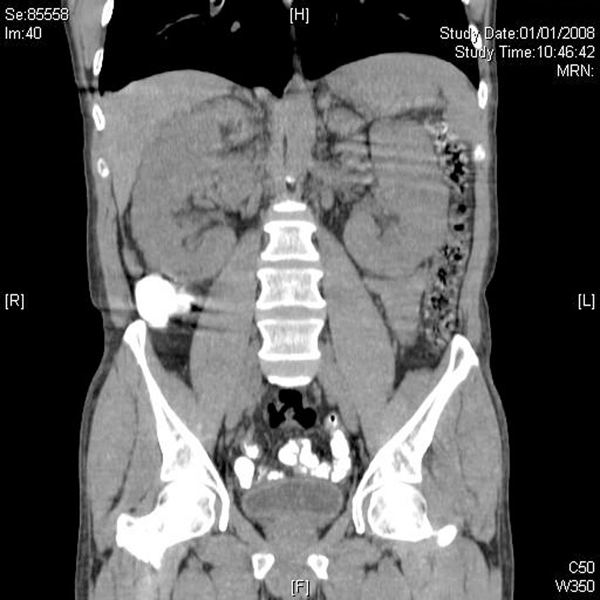
**Abdominal computerized tomographic image after therapy showing normal-sized kidneys**.

The patient underwent a five-session course of cyclophosphamide, doxorubicin, vincristine and prednisone. After this, the patient experienced considerable improvement in the function of his kidneys. Creatinine levels dropped to 1.2 mg/dl, and a repeated CT scan showed that the kidneys had reduced in length to 13 cm (Figure 6). After 6 months, we re-examined the patient. He already appeared to be in good health and had no health-related complaints. Repeated laboratory tests showed decreased globulin levels, and his blood count showed no elevation in the eosinophil level.

## Discussion

Angioimmunoblastic T-cell lymphoma represents 1% of all lymphomas. It was first described in 1974 [1] and called angioimmunoblastic lymphadenopathy with dysproteinemia. Later, when genetic unity was found in the T-cell receptors, it was categorized as one of the peripheral (mature) T-cell lymphoma (PTCL) group. This disease is usually diagnosed among men who are 40 years or older. It is characterized by B symptoms (fever of over 38°C, drenching night sweats or unintentional weight loss), lymphadenopathy, polyclonal hyperglobulinemia and eosinophilia [2]. Nephrotic syndrome in angioimmunoblastic lymphoma is uncommon and mentioned only in isolated case reports [3,4]. The median survival is about 30 months and the cause of death is usually due to infections. During biopsy, an involved lymph node will show destruction of the architecture and an infiltration composed of lymphocytes and eosinophils. Immunophenotyping will show mature T cells that are T-helper cells. Therapy is composed of doxorubicin-based regimens. Complete response rates are 64% [5].

## Conclusion

The unusual presentation of the disease consisting of giant kidneys with no nephrotic syndrome in our patient is uncommon. He also did not have the splenomegaly and skin manifestations found in half of affected patients [2]. This case shows that angioimmunoblastic lymphoma can present with huge kidneys without nephrotic syndrome. This uncommon presentation of this rare disease together with the remarkable findings on the CT scan and PET-CT make this an educational case.

## Abbreviations

CT: computerized tomography; PPD: purified protein derivative; PTCL: peripheral (mature) T-cell lymphoma.

## Consent

Written informed consent was obtained from the patient for publication of this case report and any accompanying images. A copy of the written consent is available for review by the Editor-in-Chief of this journal.

## Competing interests

The authors declare that they have no competing interests.

## Authors' contributions

OA attended the patient, collected data and wrote the manuscript. GC attended the patient and revised the manuscript. MW (head of department) attended the patient, and conceptualized the peculiarity of the case. AS revised the pathological specimens.
